# A Stimulated Recall Method for the Improved Assessment of Quantity and Quality of Social Media Use

**DOI:** 10.2196/15529

**Published:** 2020-01-28

**Authors:** Nastasia Griffioen, Marieke M J W Van Rooij, Anna Lichtwarck-Aschoff, Isabela Granic

**Affiliations:** 1 Developmental Psychopathology and Treatment Radboud University Nijmegen Nijmegen Netherlands

**Keywords:** technology use, stimulated recall, social media, well-being, qualitative research, interview, digital technologies

## Abstract

**Background:**

Social media are as popular as ever, and concerns regarding the effects of social media use on adolescent well-being and mental health have sparked many scientific studies into use effects. Social media research is currently at an important crossroads: conflicting results on social media use’s effects on well-being are abundant, and recent work in the field suggests that a new approach is required. The field is in need of an approach involving objective data regarding use where necessary and attention to different kinds of detail such as the why and how of social media use.

**Objective:**

We present a novel paradigm implementing a principle from educational sciences called stimulated recall and demonstrate how it can be applied to social media use research. Our stimulated recall paradigm implements a number of elements that can fill the gaps currently present in social media and well-being research.

**Methods:**

Objective data are collected regarding users’ social media behaviors through video footage and in-phone data and used for a structured stimulated recall interview to facilitate detailed and context-sensitive processing of these objective data. In this interview, objective data are reviewed with the participant in an act of co-research, in which details such as the reasons for their use (eg, boredom) and processes surrounding their use (eg, with whom) are discussed and visualized in a stimulated recall chart.

**Results:**

Our ongoing study (N=53) implementing this paradigm suggests this method is experienced as pleasant by participants in spite of its personal and intensive nature.

**Conclusions:**

The stimulated recall paradigm offers interesting and necessary avenues for approaching social media use research from new angles, addressing aspects of use that have thus far remained underexposed. The answers to questions such as “Why do adolescents use social media?” “In what ways exactly do they use social media?” and “How does social media use make them feel in the moment?” are now within reach, an important step forward in the field of social media use and well-being research.

## Introduction

Digital technologies such as social media have seen an immense increase in adoption and popularity. Whereas in 2005 only 10% of the United States population reported using one or more social networking websites, in 2015 this percentage had skyrocketed to 65% for the entire population and to 90% for people aged 18 to 29 years [1]. Social media enable people to be more easily connected to others all around the globe, and their potential for expansion of social networks is likely what drives these platforms’ popularity. Social media feed directly into the fundamental human need for social connection, which may be especially true for children and adolescents, who have grown up in a world in which digital technologies permeate almost every aspect of their daily lives (eg, in playing games, at school, in doing homework, chatting with friends, and even dating).

This synthesis of online and offline experiences in the lives of many children and adolescents has sparked a lot of debate among the general public as well as with researchers. Concerns about digital technologies center around screen time, since most technologies are accessed through screens that are carried around everywhere. Strong concerns have been raised regarding the effects of these screens, and social media in particular, on the well-being of youth [2,3]. As a result, many studies have been conducted to clarify (not without pressure from the lay public) what exactly the use of digital technologies is doing to youngsters’ mental well-being and development [4-10]. The debate keeps raging on, and more and more studies are added to the already large body of work on the relationship between social media use and youth well-being. Yet there is strikingly little consensus on the matter, as illustrated by two recent literature reviews [11,12]: some studies indicate a negative relationship between social media use and well-being [6,13] and others a positive relationship [5,14,15].

This lack of unanimity in the field may have to do with important methodological limitations. First, social media use and well-being research has been largely characterized by a focus on quantity, operationalized by metrics like frequency and duration [5,10,15-19]. This is problematic because such metrics do not tell us anything about the types of activities, the contexts in which they take place, and how they are experienced by users. These types of context specifics, however, seem to be what differentiates negative and positive outcomes of social media use; for instance, whether social media are used actively or passively makes a difference to users’ well-being [20]. Second, in most cases such metrics are being assessed using a method that is not particularly suited for these target variables—self-report [9,16,21-27]. Alarmingly enough, studies have shown that people are in fact notoriously bad at recalling details about their use of social media or other digital technologies [28-30]. If metrics such as duration and frequency of use are what we are interested in relative to well-being, it is vital that reliable, objective data on these behaviors are gathered rather than self-report data. Third, when self-report is used, it is generally in the context of observational studies, where no manipulation takes place [31-39], making it impossible to draw a causal inference. Additionally, when experimental designs are used, they mostly involve fabricated social media–like environments [40,41] rather than users’ personal accounts, which offer much more salient and ecologically valid contexts for studies. Also, most experimental studies in the field arbitrarily choose one type of social media platform [7,20,42-46] at the exclusion of others, often meaning outdated apps are being studied, or only one app, when in fact youth use several simultaneously. Focusing on one platform also brings forth the danger of selection bias, since there may be differences (eg, age) between user bases of different platforms that can be relevant for a study and its outcomes.

Social media might be a context that requires a radically different approach, a new methodological lens—one that is objective and accurate, while considering the unique (ie, socially salient) digital context. Thus, to extend current research on social media use and address the pitfalls present (ie, use of retrospective self-report and a focus on quantity only), we suggest that a new approach should implement objective data where quantitative measures are concerned and include a context-sensitive aspect in which attention is paid to what users are doing exactly, who they interact with, and how these specific conditions and experiences make them feel. The functions (ie, why youth use social media) of and processes (ie, in what ways, with whom, and when youth use social media) surrounding social media use have simply not been addressed by the majority of studies in psychological science. Such research questions require an ecologically valid and detailed approach that allows for quantitative and qualitative data sources, and we suggest that stimulated recall holds promise in this area.

In his original version of the stimulated recall method [47], Bloom [48] played audio from lectures versus study discussions to his students and asked them to comment on their thoughts during these events in an attempt to investigate differences in learning processes between these two forms of teaching. According to Bloom, the primary aim of the method is “that the subject may be enabled to relive an original situation with vividness and accuracy if he is presented with a large number of the cues or stimuli which occurred during the original situation.” As such, stimulated recall offers a way of investigating situations as they occur in the real world, without external influences or restraints. The method consists of two primary elements: one or multiple sources of objective information to aid the participant in recall and a qualitative, detailed interview of the participant’s recall of the event of interest. This combination of quantitative and qualitative techniques seems to be exactly the sort of approach from which the field of social media use and well-being research could benefit. The collection of objective data helps address the current unreliability of measures while the in-depth investigation of users’ activities, motives, and feelings helps to provide the detail and nuance that seems important. This new approach will ideally allow us to answer questions that are as of yet out of reach:

- Why do adolescents use social media in the first place?
- Which kinds of interactions do they experience on social media and with whom?
- What do adolescents expect from social media?
- How do these experiences make them feel?

Having discussed the origins and basics of stimulated recall, we will now present the methodology as it can be applied to social media use in young people, drawing examples from our own ongoing effort to implement this methodology in our study of social media use and well-being. This is an active study (started in April 2019) currently being conducted at the Radboud University Nijmegen, the Netherlands. Participants are students aged 18 to 25 years (N=53; 42 female) and are tested in the Bar Lab of the Behavioural Science Institute to ensure an informal atmosphere, predisposing participants to behave as they would in other public spaces rather than in a regular lab. The study was approved by the institution’s Ethics Committee Social Sciences, approval number ECSW-2019-020.

## Stimulated Recall for Social Media Research

### Objective Data Sources

A key criterion for a successful implementation of stimulated recall is access to objective data or, more accurately, data that anchor the recall to directly observable behavior. This could take the form of audio data; videotaped recordings; screen captures of activity on a computer, game console, or phone; back-end data from games or apps that log activity; and so on. These are the data collected to scaffold the subsequent interview process and provide the necessary memory. Two important data sources for a social media research application of this paradigm, video footage and in-app information, will now be illustrated using elements of our ongoing study.

#### Video Recording

To enable a naturalistic capturing of student social media behaviors, participants in our study were asked to wait for 10 minutes after having completed a task. The details of the procedure prior to the waiting period will not be elaborated on here, but we would like to note that for half of the participants it included a stress manipulation in the form of the Leiden Public Speaking Task [49]. After having completed the first phase, participants were told that “in no more than 10 minutes” the researcher would return and the study would proceed as planned. During these 10 minutes, and unbeknownst to the participants, their activities were recorded using a video camera in the hopes of capturing naturalistic social media use.

Whether we would be able to capture smartphone behaviors of interest depended to a large extent on the camera setup. The best solution ultimately involved a camera installed right above the participant’s seat, which has provided us with good and reliable footage (ie, the participant could change poses, but this would affect the quality of footage only minimally) of the participant’s phone in all of the cases so far. Participants were always tested in the Bar Lab room seated at a table positioned directly under the camera. To ensure that participants would not get up and walk around the room (and thus leave the camera’s field of view), they were asked to remain seated while the researcher was gone to ensure a steady signal from the physiological equipment (which, in reality, was robust to movement).

**Figure 1:**
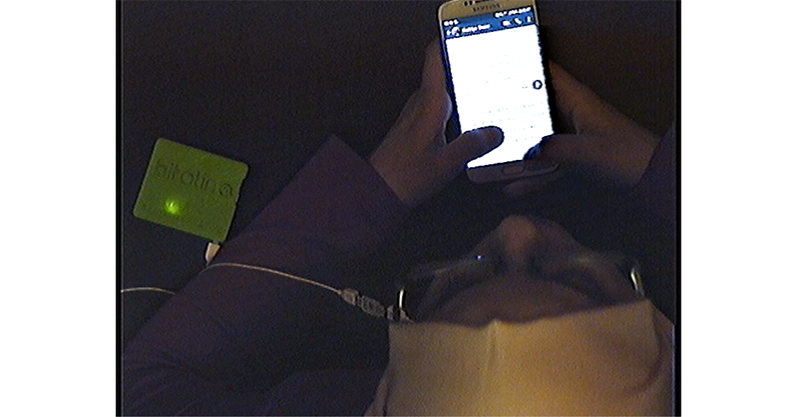
Screenshot from the video recording of one of our pilot participants.

The dome-shaped camera was able to rotate on its axis, tilt, and zoom in and out, as well as adjust focus to points nearer or further away in space, allowing us to sharpen or blur the image as necessary. The camera was controlled by the researcher from a control room next to the study room where the participant was waiting. The focus point of the camera could be controlled in such a way that the participant’s smartphone screen (if used by the participant) was visible but no text could be read from the screen to guarantee the privacy of the participant and any people whose information may have been featured on the screen. Similarly, images were always blurry, and, although shapes could be made out, any people featured on the participant’s screen could not be identified. What these recordings did enable us to see, however, was which apps the participant was using and what the participant was doing in these apps (eg, just scrolling, typing text, liking a post). See Figure 1 for a screenshot of a pilot participant’s recording. On this screenshot, for instance, we can see that the participant seems to be typing a message in WhatsApp, judging by the layout of the app visible on the screen. No preprocessing of the video footage is required before use in the interview, meaning that the interview can take place almost directly following the monitoring/waiting phase.

#### In-Phone Information

While the video recordings provide valuable information about participant activities, in a number of cases the recordings alone were not sufficient to capture our desired level of detail regarding participant phone use. For instance, very brief actions such as hitting a Like button could sometimes be harder to identify with certainty given the blurriness of the video image. In other cases, the layouts of apps were sometimes similar or even unknown, meaning that it could be hard to pinpoint exactly which app was being used. Although looking at what the participant was doing on their phone with their fingers (eg, typing, swiping, tapping) could help distinguish between certain apps that otherwise look quite similar, an extra source of information could be called upon: the participant’s own phone. Such information can always be called upon in the moment itself and does not require preprocessing.

**Figure 2:**
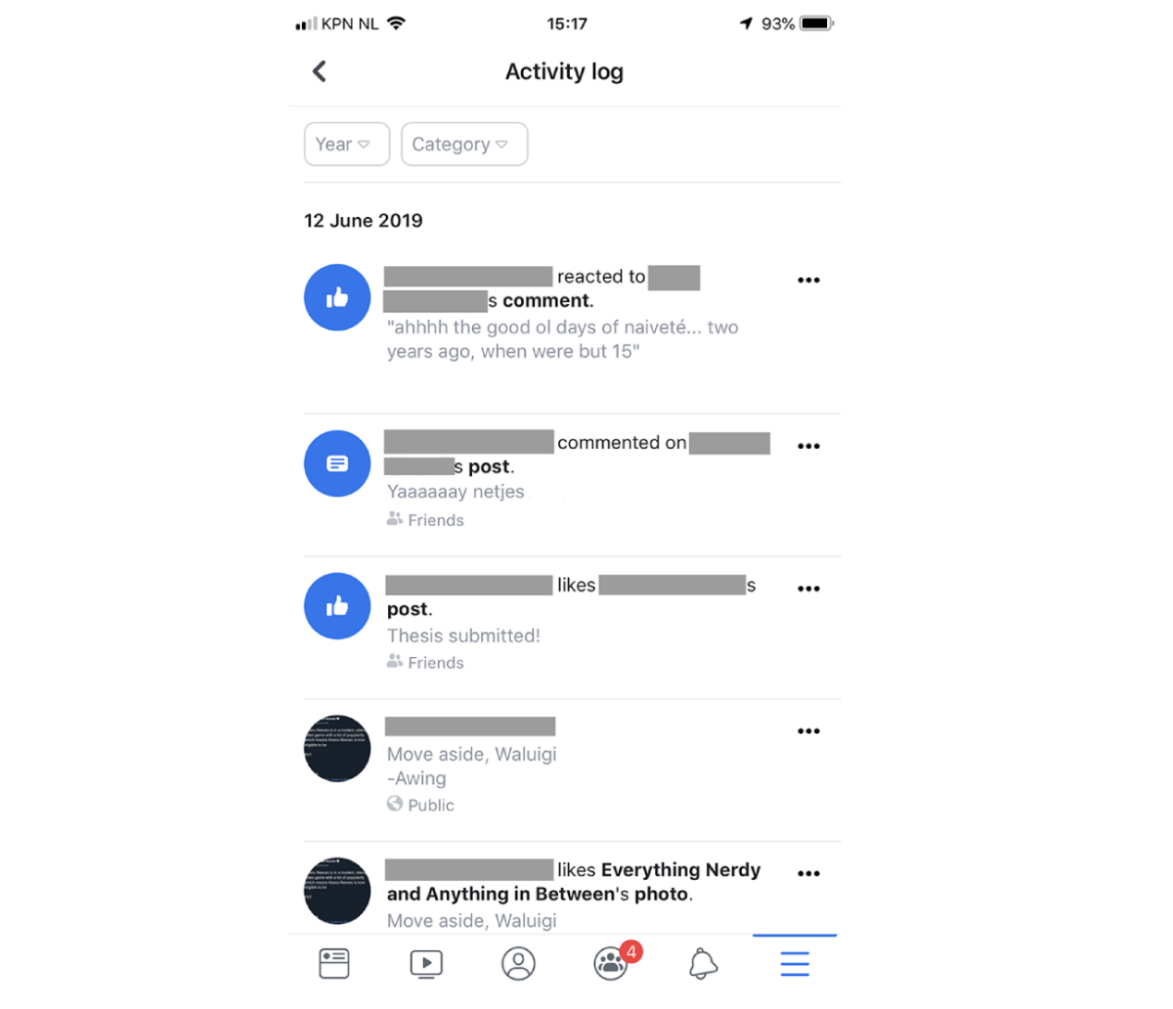
Screenshot from Facebook app illustrating the activity log.

First, if there was uncertainty about what sort of action a participant engaged in, they were asked to open up the social media app and navigate to the activity log or equivalent. Most social media apps contain such an overview of user behavior in the app, although not all of them will refer to this overview as an activity log, and in some cases, information may be scattered over a number of places within the app. For instance, Instagram has an overview of the posts a user has liked, if you dig deep enough, but Instagram does not offer an in-app overview of any comments the user may have posted (one could, however, use the less instantaneous Download My Data functionality if the comments were of particular interest). Luckily, the act of commenting could easily be identified on the video recording since the participant was typing. Facebook, on the other hand, does include comments in their overview of the user’s activity, although their activity log is similarly hard to find for an inexperienced user. See Figure 2 for a screenshot of Facebook’s activity log. This overview can be helpful for determining which data one can and cannot access in case a similar paradigm is implemented in other studies. Knowing beforehand what sorts of reliable (ie, objective) data can be accessed is paramount for study success, since the stimulated recall hinges on data to aid the participant in accurately recalling thoughts and feelings about the activities of interest.

**Figure 3:**
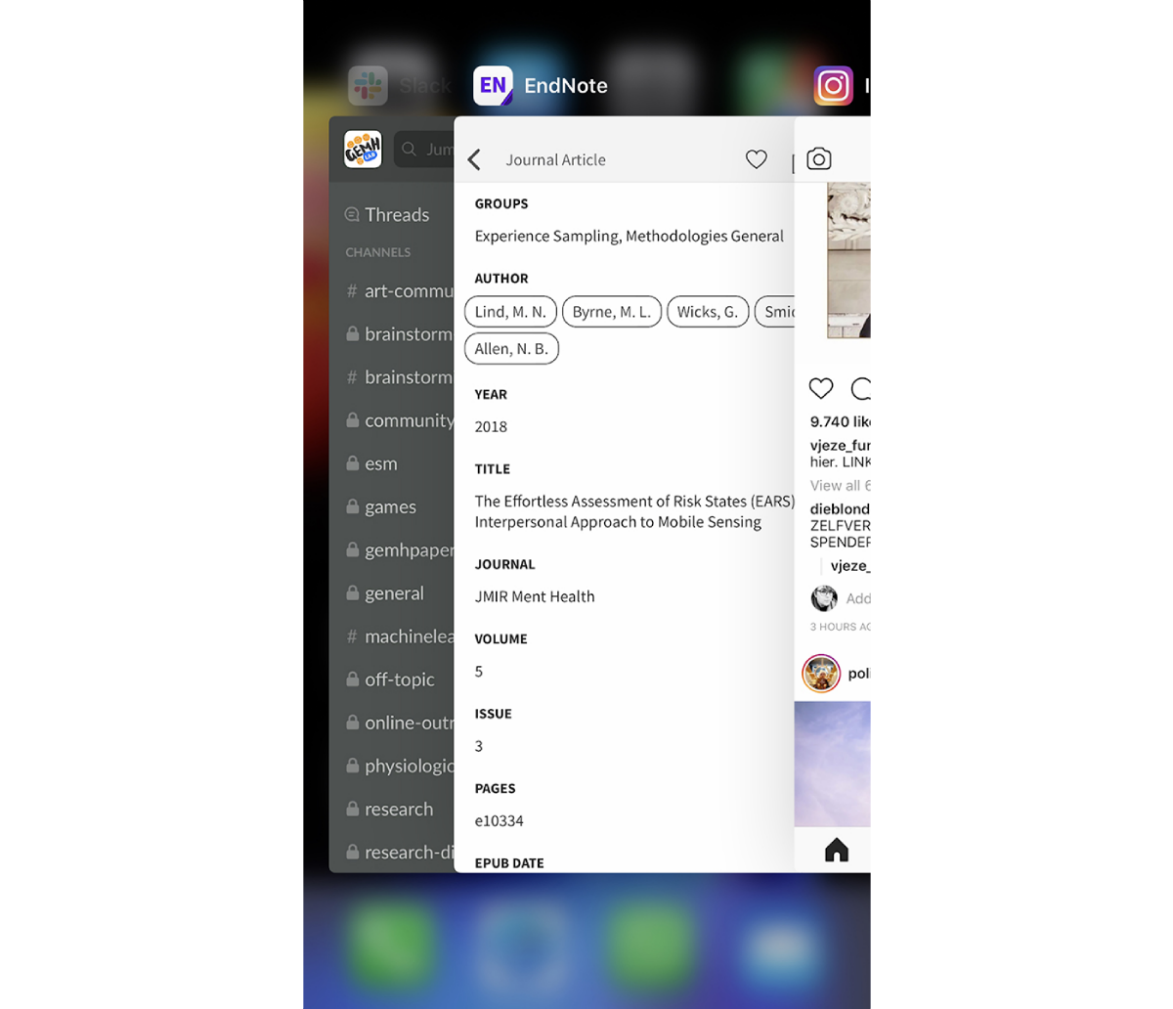
Screenshot of the opened apps overview on an iPhone.

Second, if there was uncertainty about what sort of app the participant was using, we attempted to retrieve this information using the overview of currently opened apps (see Figure 3 for a screenshot of what that looks like on an iPhone). To make sure that in such a situation we would not be faced with a sequence of apps that had been used days ago (but never closed) rather than in the 10 minutes of the waiting period, we followed a standardized procedure. During the setup of our physiological equipment used to measure participants’ electrocardiography (which we incorporated in the study to be able to check whether the stress manipulation had indeed worked), we told the participants that we would need them to turn their phones off and on again so we could do “signal calibration checks” in between to ensure that the phone’s signal would not hamper the physiological data collection later on in the study. This meant that we could check, if necessary, which apps in the opened apps overview of the participant’s phone were opened and used during the monitoring period and not before; although the app overview remains unchanged even after restarting the phone, any apps being used before the restart will need to reload when accessed from within this overview. That way, we had a way of checking whether an app was used during our study or before it. The “turning phone off and on again” request additionally meant that we could subtly check whether participants had their phone with them and ensure their phone would be near them when the waiting period arrived.

### Stimulated Recall Interview

Whereas the use of objective data sources addresses the lack of reliable information regarding user activities, the stimulated recall interview tackles the lack of attention to the how and why of adolescent social media use while incorporating the collected objective data. There are a number of important elements to the successful application of such an interview in a social media research context. First, it is important to acknowledge there are users of social media (especially the younger user base) who engage with social media by sharing relatively personal details about their daily life with friends or family. In order for an interview to be successful, trust needs to be established between researcher and participant because information discussed in the interview, namely about what is put out on social media, can be personal and sensitive. We propose that a powerful way to establish this trust is to authentically recruit the participants’ own intrinsic curiosity and generosity in the interview by asking them to join the researcher in a brief moment of co-research. As in participatory research [50], we clearly explain the general goals of our study, what kinds of data have been gathered, and how they will be used to aid in the interview. This is an important step toward eliminating any unease the participant may experience when asked to share personal details, thoughts, and feelings. Second, the interview needs to be structured and standardized across participants. Explaining the structure of the interview will not only help put the participant at ease if necessary, it will also enable the participant to be the best co-researcher they can be; if they know what the researcher is interested in, they will be best able to help and contribute. Good structure and standardization of the interview, however, does not only have to do with the fact that such interviews can be very data-rich. Thanks to a structured approach, the participant will feel there is a particular method and consistency to how personal details are being collected and handled, which will further contribute to a good relationship during the interview. Ultimately, a better researcher-participant relationship will lead to better insights into participants’ behaviors and thought processes.

In our study, participants were debriefed and told the true purpose of the study after they had completed the monitoring period in which their activities were recorded. It was explained to participants that the researcher would like to use the remaining study time to conduct a structured and detailed interview about their social media behaviors and experiences, if possible with the aid of the participant’s phone and the video recording made during the monitoring period. If the participant gave consent at this point, the study continued and the participant was interviewed following our interview protocol and with aid of the data. If not, the participant was thanked for participation so far and told that the study was ending there. Interestingly, only one of our participants so far has withheld consent for the use of video footage, suggesting participants are interested in sharing their data with us and gaining insight into their own behaviors.

**Figure 4:**
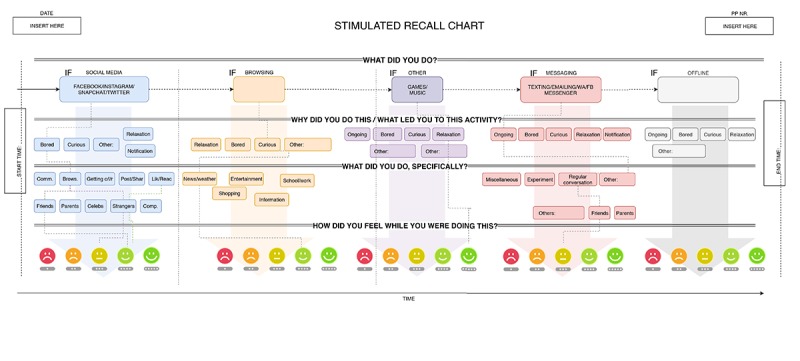
Schematic interview chart used to aid in the stimulated recall interview.

The goal of the interview was to gain insight into (1) what adolescents do on their phones, with increased specificity when it comes to social media, (2) why adolescents engage in these activities (according to them), (3) who (or whose information/posts) they encounter and interact with on social media, and (4) how these activities make adolescents feel in that moment. As such, there were a number of layers to each activity/experience we explored in the interview, and to ensure a consistent structure across participants, we developed a scheme to aid us in conducting these intensive and often personal interviews (Figure 4). By making clear to the participants that despite the personal nature of our questions there was a structure to our method, we hoped to not only facilitate data processing afterward but also predispose participants to cooperate in the interviews (only one participant out of the 53 tested so far has withheld consent for the stimulated recall interview; for more about feasibility research see Feasibility and User Research). Additionally, the interview was audio recorded for future reference and potential in-depth analyses.

During the stimulated recall interview, the video recording of the participant was viewed by the participant and researcher together. At the onset of each new major activity, the researcher would ask the participant questions according to the interview scheme (Figure 4), pausing the video when necessary and completing each of the interview layers (indicated by the horizontal layers in the interview scheme) before moving on to the next major activity. If these behaviors were on the phone, the end/start of a major activity was signified by switching to another app.

The way the interview is structured, going through the layers outlined in the interview scheme, lends the stimulated interview implemented in our study affordances that are especially important for the field of social media (and well-being) research. First, use of video footage of the participants’ activities allows us to consider time; research has indicated that we cannot expect people to accurately recall what was done [29], let alone accurately recall in what order. With the video footage as a foundation to construct our image of people’s smartphone and social media behaviors, we can pinpoint time stamps to in-app behaviors (eg, liking a post, reading/scrolling, typing a message, browsing the internet), app switching behaviors, and behaviors like switching from using the smartphone to doing something offline (eg, reading). This allows for the measurement of relatively unexplored variables such as behavior pattern dynamics (eg, whether participants engage in long bouts or short bursts of different activities or whether activities are triggered by incoming notifications or self-initiated), and importantly, allows for accurate assessments of the duration and frequency of behaviors. When viewing the video footage, a first look is taken at the major activity (eg, Facebook). The participant is asked to describe why—to the best of their recollection—they started engaging in this major activity in that moment. Participants may indicate they had a specific goal in mind (eg, “I wanted to look up the profile of a girl a friend mentioned”) or they were simply bored and they always go to Facebook when bored. Next, the footage of the major activity is re-viewed and dissected into subactivities done by the participant. For instance, one participant may have scrolled the news feed, liked a number of posts, and commented on one of those posts, whereas another participant might stick to only scrolling the news feed. These subactivities are noted for each major activity. Note that the same major activity may occur multiple times, since people often switch to other apps but then come back afterward. Thanks to the video footage, we are able to capture such repetitions and any differences in behavioural pattern shapes that may occur between participants, giving a much needed, detailed view of how exactly adolescents interact with their phones and social media.

A second advantage to this method is that the interview setting allows us to put the social back into social media research and offers a much more in-depth assessment of whom social media users are engaging and interacting with on these platforms. Social media are, of course, meant to enable social interaction between people all over the world. Additionally, the types of people (eg, family members, friends, acquaintances, strangers, celebrities) users come across on social media may differ vastly depending on the platform. These intricacies of social media use have thus far been ignored in many studies of social media and well-being and can now be addressed in the stimulated recall interview. For each type of subactivity within a major activity (eg, liking posts in a particular Facebook session), we ask participants what type of other people were involved in the subactivity (eg, “What kinds of people posted the messages that you liked?”). The participant is offered a number of suggestions (eg, “Were these messages posted by a friend of yours or by a stranger maybe?”) and if possible, the participant’s phone is used during the process so that the participant can accurately recover who, for instance, posted the messages they liked. After the participant describes what these people are to them, one of 6 categories is written down next to the subactivity involved: friends, family, romantic partner, acquaintances, strangers, or celebrities.

A final advantage to our approach is the in-depth, qualitative nature of the data we can collect. This includes what users did on social media and who they interacted with but also how these behaviors and experiences made them feel. Asking someone to describe the experience of reading a friend’s post is hard to do without the context of the post itself, and although there have been studies attempting to artificially recreate such contexts [51], this method provides a more reliable, ecologically convincing account of social media interaction as they emerged spontaneously in a naturalistic context. In our stimulated recall interview, we do not ask participants to elaborate on their feelings for every post they read or emoticon reaction they gave but, given their stimulated recollection of what they read or did, elaborate on their feelings and experiences for the types of activities within the major activity at hand (eg, for scrolling and viewing posts within Facebook). For each of these activities, they indicate with a smiley how they felt in a general sense (on a 5-point Likert scale; see the smileys on the bottom of the interview scheme in Figure 4). After participants indicated which smiley best reflected their feelings for a given activity, we asked participants to briefly describe why they chose this particular smiley and explain how they felt specifically. For instance, if a participant indicated that they felt moderately negative (smiley 2) while scrolling/browsing posts on Facebook, they might say this had to do with the fact that they saw a lot of negative news and it made them a little sad. For this emotional layer of the interview, no categories are used (in contrast to the other layers of the stimulated recall interview). Instead, key words used by the participant when describing how they felt and why are written down. Given that feeling ratings and descriptions are category-based (eg, for scrolling/browsing posts during this particular session of Facebook) rather than per every post they encountered, it may happen that participants report having felt positive emotions for one post and negative emotions for another post. In such cases, participants are asked to select the smiley that most accurately reflects their average feeling about the posts (for instance, by selecting the neutral smiley). The details can always be reflected in the feeling description: it might, for instance, say “It was nice because I saw a funny post, but also sad because a friend of mine had some bad news.” While the feeling rating in the form of a smiley is a compact measure, the description element within the feelings layer of the interview plays a vital role in truly finding out how adolescents feel during their social media visits and for which reasons.

### Stimulated Recall Chart

**Figure 5:**
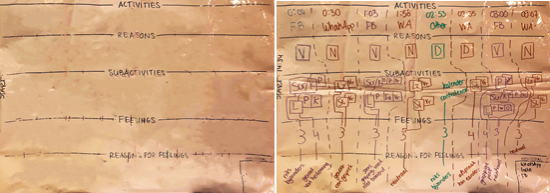
Left panel: empty whiteboard foil sheet prepared for the stimulated recall interview with the basic skeleton already drawn on it. Right panel: completed example of the stimulated recall chart. Major activities are indicated in the top row; reasons to engage in these activities are indicated in the second row (N: due to a notification; V: boredom); specifics surrounding the activities are indicated in the third row (Scr/K: passive viewing; L: like); and the fourth and fifth rows contain information about participants’ feelings during these activities (on a scale from 1-5, with a brief description).

The stimulated recall interview yields a rich body of information and data about participant behaviors and experiences. When developing the design of the study and particularly the stimulated recall interview, we developed a standardized chart to formalize all the information coming forth from the participatory interview. After a couple of iterations, we landed on a layout that closely resembles the schematic interview chart (Figure 4). At the start of the interview, a sheet of whiteboard foil with the general skeleton of the chart already set up (Figure 5, left panel) is explained to the participant, and we say we would like the interview to be collaborative and have the participant engage in the research that happens during the interview, together with the researcher. As the interview progresses, we fill out the sheet together, which results in an information-dense but highly structured visualization of the participant’s monitoring phase (Figure 5, right panel).

Depending on the activity level of the participant, multiple sheets may be used to capture all of the activities they participated in during the testing phase. After the interview, photos are taken of the sheets to be stored on our secured data servers, and the whiteboard foil sheets are wiped clean, removing all but the initial skeleton of the chart, ready to be used for the next participant.

## Feasibility and User Research

### Monitoring/Waiting Period

Participants were kept unaware of the aim of the study to allow for an optimally naturalistic assessment of adolescents’ activities during the waiting period. We expected, from personal and anecdotal experience, that adolescents would pull out their smartphones when asked to wait, and indeed, a pilot we conducted with this method (N=8, all female) indicated that the smartphone was participants’ go-to activity. We found an overwhelming display of smartphone use despite the participants’ bags being close enough for them to engage in other activities they may have had brought with them (eg, reading a book). This latter aspect of the design (ie, bringing the participant’s bag close) was also piloted, since we wanted to give the participant the feeling they could do whatever they wanted (as long as they remained seated) while not diminishing our chances of capturing the behavior of interest (ie, smartphone and social media use). Ultimately, 100% (n=8) of the pilot participants used their phones. Moreover, very few engaged in nonsmartphone activities; only one pilot participant engaged in one offline activity (ie, not involving the phone) in addition to a number of smartphone activities. This participant put away their phone after a couple of minutes and spent the rest of the time investigating the room. Interestingly, this was an older participant (age 54 years), which might explain the difference in behavior compared with our other pilot participants, who were all within the age range of interest (18-25 years). As anticipated, social media were used by nearly all of our pilot participants during the waiting period (again with exception of the participant aged 54 years); in our study, this has been true for all but 9 participants.

Whether the waiting period would allow us to capture the behavior of interest was not the only reason for piloting our paradigm. The duration of the waiting time proved to be a nontrivial issue. Prior to the start of the pilot, durations of 10, 15, and 20 minutes were discussed. We wanted to ensure the participants had enough time to exhibit the full range of possible activities (within the constraints of our study): we didn’t know whether participants would go straight to more leisurely activities or attempt to do study-related work first (given that our participants were likely to be students). On the other hand, we did not want the waiting time to affect participants negatively (since waiting too long can be perceived as annoying and might affect the mood). We settled on 15 minutes for the pilot and discovered two issues that directly affected our study. One pilot participant opened an app such as Netflix shortly after the researcher left and continued to watch streaming content for the rest of the waiting period. This, of course, posed a problem for our paradigm, given that we wanted to maximize our chances of capturing social media behaviors. Also, we quickly realized that a 15-minute waiting period (which by definition yielded a 15-minute monitoring video) significantly prolonged the duration of the stimulated recall interview that followed. After a couple of pilot participants, we found that 5 minutes of monitoring footage would take approximately 15 minutes to interview with the participant, bringing the total interview time to 45 minutes in the case of a 15-minute waiting period. These two factors (ie, the predisposition to watch streaming content when told one has to wait for 15 minutes and interview length being a multiple of the waiting period duration) led us to pilot the remaining participants with a monitoring duration of 10 minutes instead. The remaining pilot participants did not engage in streaming series or films, and the interview duration was brought back to 30 minutes, which proved to be more palatable for the participants given the intensive nature of the interview.

### Participant Experience

As discussed earlier, the stimulated recall interview could be considered intensive due to the interview’s duration and the level of detail of recollection required. To ensure that stimulated recall paradigms like ours can be used in further studies, experimenters need to make sure the burden on the participants is not too heavy and participants are willing to share their often personal information. Throughout the final phase of our pilot (after the design changes discussed earlier had been consolidated) we asked participants off the record what they thought of the interview and if they would change anything about it, and none of our participants indicated that the interview was too burdensome.

**Figure 6:**
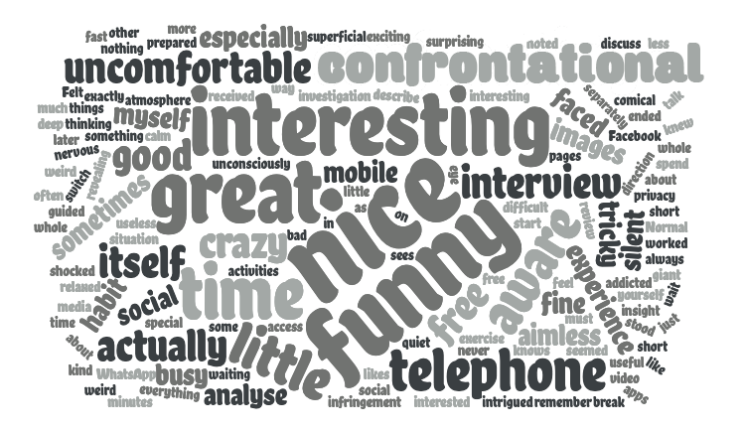
Word cloud depicting words used by participants (n=53) in response to the statement “Please describe how you experienced the stimulated recall interview.” Larger words represent more frequently used words. Font darkness is for decorative purposes only and does not represent other aspects of these data.

Additionally, although they were often surprised that they had been filmed without their knowledge, all of our pilot participants (n=8) were very willing to share with us how they had spent their time on social media, despite the personal nature of these experiences. We believe that this had a lot to do with the researcher’s attitude: transparency is very much valued, even if it is after the fact, and including the participant as much as possible in the process (rather than just using their data) is likely what predisposed our participants to engage in open-hearted conversations during the interview. The emphasis on co-research has also made it easier for our researchers to conduct such personal interviews (rather than feeling like intrusive voyeurs). With the exception of one participant in our currently running study (n=53) who withheld consent for the use of the monitoring footage, we have found the same as in the pilot: if approached in an open way with genuine interest, participants are excited to explore their own social media activities and experiences with us. For this study, we have decided to include two questions in our debrief measure regarding participant experience in the stimulated recall interview. First, participants were asked to describe in a few sentences how they experienced the stimulated recall interview. Second, participants were asked to indicate on a scale from 1 (very unpleasant) to 10 (very pleasant) what they thought of the stimulated recall interview. Based on the currently collected sample of participants (n=53), we can say that on the whole participants found the method rather pleasant (mean 7.46 [SD 1.27]). The words most used (Figure 6) to describe the stimulated recall method were nice (15), funny (11), interesting (8), great (8), and confrontational (7). These data seem to indicate participants were engaged and enjoyed the process, even though seeing themselves engage in activities that some participants described as not particularly productive was considered confrontational by some.

## Considerations

The application of stimulated recall to social media research is showing promise in helping researchers investigate the functions and processes surrounding social media use by adolescents, while guaranteeing data are reliable. This addresses two important issues currently discussed: the use of self-report for assessment of quantitative aspects of social media use and the lack of attention to the contexts in which social media are used. With the stimulated recall chart as a rich source of data, research questions can be addressed that were out of our reach before. For instance, we will be able to examine students’ key motivations for using social media and determine whether use of social media is fueled by the apps themselves (ie, because notifications come in) or by a form of intrinsic motivation. We will also be able to examine in much greater detail what exactly students spend their social media time on and what kinds of social ties (eg, friends, family members or celebrities) they come into contact with on these platforms. Last, and most elusively, we will be able to get a sense of how all of these aspects of social media use intertwine and culminate in user experiences; how social media use makes people feel likely has to do with the specifics of their use, and we will be able to link the affective experience of social media use to specific aspects of social media use thanks to data gathered through the stimulated recall interview and chart. Importantly, we are not suggesting that stimulated recall should be used exclusively in future research into social media use and well-being. This method, in principle, lends itself well to many other fields, whether the study subject concerns other kinds of new media use (eg, video gaming) or consumer behavior. We suggest that, depending on the research questions at hand, the field can benefit from combining methods such as experimental designs with a stimulated recall approach because this approach is largely content-agnostic: any type of objective data can be used as long as it aids participants in their recall of certain events or experiences. Such multimethod paradigms, if constructed in ecologically valid and reliable ways, will be important steps forward in the field of social media use and well-being.

Despite the clear affordances of this new method, limitations and considerations should be addressed by anyone implementing a similar paradigm in the future. First, the method we have presented is restrictive in that it relies on a setup with cameras. As we found during the piloting phase, placement of the cameras is crucial to the stimulated recall interview because objective sources of information are required to aid the participant in their recall of events. While one could think of nonlaboratory situations (eg, malls, cafes) in which multiple cameras could either be installed or are already present, such contexts bring with them other problems. Privacy issues would arise concerning all other people present in that space who are also captured by the cameras. Recording footage of people without prior consent could be considered ethically unacceptable by institutional review boards. Hence, the stimulated recall method as we have described it here is more suited to controlled settings than field contexts, although the latter might be possible if additional measures are taken to protect the privacy of everyone involved.

Second, stimulated recall interviews do not capture dynamics of behavior over longer periods of time. The monitoring duration is restricted by the following length of the stimulated recall interview and the fact that participants need to wait in one specific lab room. This means only a relatively short period can be captured. To somewhat alleviate this limitation, researchers could adjust the level of detail addressed in the stimulated recall interview (and the data following from that interview); if fewer aspects of behaviors are of interest (eg, only whether something was posted or shared on social media), the stimulated recall interview can be cut short significantly. The stimulated recall interview is a relatively time-intensive form of measurement and, although the timeframe assessed may stretch to 30 minutes or even an hour if that level of detail is needed, the method is simply not suited to assess behaviors (and changes in behaviors) on a time scale that spans multiple days or even weeks.

Third, we have not asked participants to use their phones during the waiting period, and their use of phone and social media can therefore be considered quite natural. However, it should be noted that in our setup, participants are not offered alternative choices of activities, which makes the situation different from usual private life, in which one may be able to choose from using their phone, or reading a book, or watching television. We feel that our setup sufficiently resembles and represents many spare moments in everyday life when roaming public spaces, such as waiting for a friend at a café or riding public transportation.

Fourth, although we have tried to incorporate the best objective data sources available to us in social media contexts (ie, external recordings of the phone and in-phone information), there is still a great amount of data stored within apps and on company servers that remain inaccessible to researchers, mostly due to restrictive data policies asserted by large tech companies. Such data, were they accessible, would be able to shed light on more extended versions of questions that are now assessed using this paradigm. For instance, patterns of behaviors could be assessed over longer periods of time because they could be passively sensed rather than recorded actively in the lab. Although the qualitative, experiential aspect of such behaviors cannot optimally be addressed remotely and will still require an interview with a researcher, reliably measured changes in objective aspects of digital technology use behaviors could be addressed. Additionally, changes in mood could be measured through a complementary experience sampling setup [52], which could then be linked to specific and reliable behavioral data. Efforts toward passive sensing of smartphone use are actively being made [53], but hurdles remain: the fact that many companies do not make their data available to researchers continues to hinder researchers in reliable assessment of users’ behaviors. Related to this inaccessibility of data is the fact that users’ privacy needs to be ensured no matter what, and, fortunately, efforts toward transparent and privacy-safeguarding protocols are already being made.

Last, we would like to address some ethical considerations pertaining to this kind of protocol. What adolescents do on their phones and on social media can, of course, be highly sensitive. Recording people without their knowledge and consent, especially as they engage in activities generally considered private, is not ideal from an ethical point of view. However, we feel such a lie by omission is necessary to ensure participants display naturalistic behaviors. This type of design is similar to any requiring some form of deception that is later revealed to research participants. However, we take these ethical considerations seriously and have taken steps to minimize concerns and ensure participants provide informed consent as soon as possible during our procedure. Participants are asked for explicit consent a second time, between the collection and use of the data; they are clearly told that they should feel free to withhold consent; they are also assured that they will receive compensation for their time in our lab no matter their decision.

Finally, it is worth mentioning that the nature and extent of precautions taken with regard to storage may depend on the characteristics of the data collected. As we mentioned earlier, participants’ faces are not shown in our video footage, and no text or people can be discerned on their phones. However, if the exact content of apps and messages can be read and contacts’ faces are recognizable, these factors bring with them new challenges such as ensuring the privacy of those contacts is being guaranteed or their consent obtained.

## Conclusions

More attention should be paid to the qualitative side of digital technology use, since frequency and duration metrics can only tell us so much and that seems not to be enough [54]. We have presented a novel paradigm that can be implemented in digital technology (eg, smartphone and/or social media) use research. The application of stimulated recall to these contexts allows us to not only more reliably assess user behaviors but also to address how users think and feel while interacting with digital technologies. We hope that through this paradigm, new insights into people’s digital lives can now be gathered in contexts that are ecologically valid and honor the spontaneous and automatic nature of the behavior of interest. Although there are limitations to the stimulated recall paradigm, depending on the question of interest, these can be justified by the insights this method can provide. With more concrete and detailed information about the ways in which users engage with social media and how this makes them feel, researchers in this field will be able to design better studies in the future (whether they be experiments, diary studies, or observational studies) that are ecologically valid and maintain the context sensitivity necessary to capture or create naturalistic behaviors. Ultimately, we hope this new approach will help researchers work toward a better understanding of why, with whom, and how exactly users interact with digital technologies such as social media and how these experiences affect users’ mental health and well-being.
